# Progressive Keratoconus Treatment with Transepithelial Two-Step Phototherapeutic Keratectomy Combined with Corneal Crosslinking (CXL): Clinical Outcomes and Postoperative Management Including Potential Complications of the Modified Athens Protocol Designed for US-Approved Excimer Laser Specifications †

**DOI:** 10.3390/jcm13237024

**Published:** 2024-11-21

**Authors:** Anastasios John Kanellopoulos, Alexander J. Kanellopoulos

**Affiliations:** 1 Ophthalmology Department, LaserVision Ambulatory Eye Surgery Unit, 11521 Athens, Greece; alexandrosjohnk@icloud.com; 2 Ophthalmology Department, NYU Grossman Med School, New York, NY 10016, USA

**Keywords:** ectasia, keratoconus, cornea crosslinking, excimer ablation, PRK, Athens protocol

## Abstract

**Objectives:** To report a novel application within the USA of excimer ablation for the normalization of central corneal refractive irregularity, combined with higher fluence CXL in the effective management and visual rehabilitation of progressive keratoconus. **Methods:** 17 consecutive cases with progressive keratoconus were treated with corneal surface excimer laser ablation normalization using topography-guided (Contura) myopic ablation for customized corneal re-shaping with a 6 mm optical zone. The epithelial removal was accounted for by adding a −2.75 diopter correction to this topography-guided normalizing surface ablation followed by a second wavefront-optimized hyperopic excimer treatment of +2.75 diopters also with a 6 mm optical zone. The two sequential excimer ablations applied on the intact epithelium were followed by corneal crosslinking (CXL). Visual acuity, refraction, and keratoconus documentation via keratometry, topography, and pachymetry, as well as endothelial cell density were evaluated over 36 months. **Results:** Keratoconus stabilized in all cases. The severity and stage of keratoconus determined by the Amsler–Krumeich criteria improved for the OD from an average of 2.2 to 1. The median UDVA showed marked improvement at one-year follow-up (all values in LogMAR), from 0.8 preoperative to 0.3 at 12 months, and was stable through the 3 years at 0.3. The median CDVA increased from 0.5 to 0.1 at 1 year and was stable at 0.1 at 3 years. The average minimal corneal thickness decreased from 466 μm to 396 μm, as recorded the first year postoperatively, and then slightly increased to 405 μm at the 3-year follow-up. **Conclusions:** We introduce herein the initial clinical data for the use of a novel, off-label therapeutic excimer laser surface ablation application. It was designed to perform both epithelial removal and anterior corneal stroma reshaping and combined with CXL to apply the Athens Protocol CXL with US excimer laser-approved specifications.

## 1. Introduction

The last two decades the initial Dresden CXL protocol has changed the treatment paradigm for the management of keratoconus and corneal ectasia [1,2,3,4,5,6,7,8,9,10,11].

The technique has evolved to include higher fluence CXL and in some cases the use of therapeutic customized surface ablation to facilitate riboflavin absorption into the corneal stroma and likely also into the actual CXL process.

Our investigative team has introduced the technique called the Athens Protocol CXL [12,13,14,15] and, subsequently, many other clinicians globally have applied the same or modified techniques with comparable outcomes [16,17,18,19,20,21,22]. Globally, outside the US, the first and most common technology that has been employed as an adjunct to CXL is based on the topography-guided platform using excimer lasers initially from Alcon/WaveLight (Erlagen, Germany) and later from Schwind Amaris (Kleinostheim, Sweden), IVIS (Taranto, Italy), and Nidek (Gamagori, Aichi, Japan).

Our clinical team has had the opportunity to employ CXL for keratoconus and corneal ectasia both in Europe, as noted above, commencing two decades ago, and subsequently, in the United States, once CXL became approved in 2016, the same year topography-guided treatments for primary myopic eyes became FDA approved as well. Keratoconus and ectasia patients advised in the past to be treated with the Athens Protocol outside the US—usually in Canada, or even by travelling to Europe—were now able to have this treatment as an off-label excimer laser and CXL application in the United States. The clinical work described herein was designed to overcome the practical limitations—in existence even currently—regarding the application of Athens Protocol-related surface, normalizing, and excimer surface ablation in the United States. Of note here is that the Alcon/Wavelight excimer laser was the only commercially available and FDA-approved device to offer topography-guided treatments within the United States when these treatments were performed (2017–2020). The device, within its US-approved specifications, only allows for treatments with a 6 mm minimum optical zone for the topography-guided platform (Contura, Alcon/Wavelight, Erlagen, Germany). The spectrum of refraction treatment along with the topography-guided normalization is limited to zero refraction, myopic, or myopic astigmatic spherocylindrical treatment. No hyperopic or hyperopic astigmatic treatments are feasible within the US specifications for Contura treatments as of yet. Additionally, there is no option for a PTK (phototherapeutic) mode, which could be used for epithelial removal. A review of the technique and technology that our clinical team and other global clinicians have employed is described and summarized in our editorial by invitation in the Journal of Cornea [23].

In this study, we report a consecutive case series of this modified technique in order to be able to perform the Athens Protocol CXL within the excimer laser treatment specifications available in the United States currently.

## 2. Methods

This is a retrospective review of a consecutive case series. All patients had also initially provided their informed consent and approval for clinical data analysis. This study is adherent to the declaration of Helsinki. The retrospective review of data was approved as a study by our institution’s ethics committee (Laservision Ambulatory Surgical Unit Ethics Committee). This consecutive case series included patients with established keratoconus that was found to be progressive and intolerant to contact lens use.

All cases were adults aged 18 years or older at the time of the initial assessment.

All cases had minimal corneal thickness of at least 400 μm, as measured by anterior segment OCT (RTVue XR Avanti, Optovue Inc., Fremont, CA, USA. Software version: Version 2016.1.0.26).

Excluded from this consecutive case series were patients that had any of the following ocular conditions contraindicating this intervention: previous corneal hydrops, corneal scarring, known diagnosis of herpetic corneal disease, and connective tissue systemic disorders predisposing to corneal scarring following surface excimer ablation to also include a history of known keloid skin formation.

All patients received detailed informed consent regarding the advantages and potential disadvantages that combining a therapeutic topography-guided surface ablation with higher fluence CXL may have. All cases were evaluated by expert optometric-associated staff and followed for up to three years postoperatively. Descriptive statistics were used to present these data in a grouped form.

**Figure 1:**
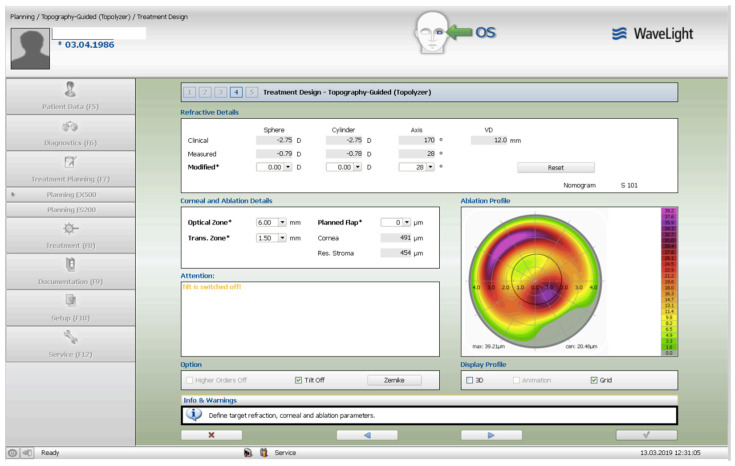
The Contura treatment designed with zero refraction.

**Figure 2:**
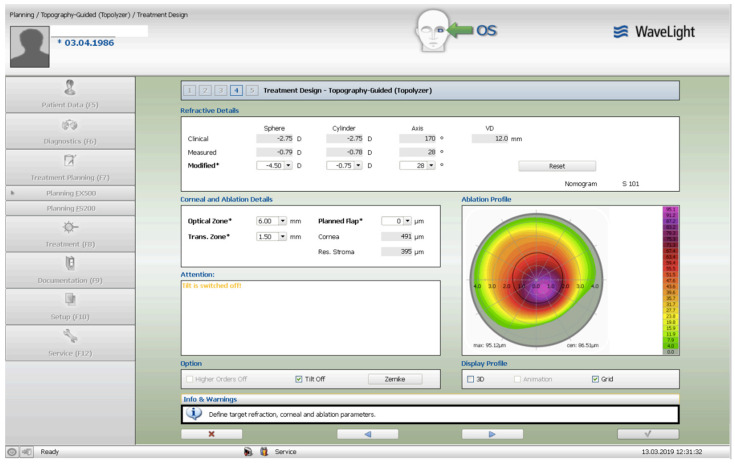
The same refraction with an additional −2.75 diopters of myopia to account for central epithelial removal. Once the Contura treatment was designed in a similar fashion to our Athens Protocol cases outside the US, an additional −2.75 diopters of myopia was added to the refractive error planned to be included in the topography-guided platform—usually 1 to 2 diopters of myopia and up to −2.5 diopters of astigmatism. Then, the same absolute number of +2.75 was used for the WFO hyperopic treatment to follow the same optical zone of 6 mm—seen next in Figure 3. The goal was to account for epithelial removal along with the therapeutic topography-guided surface ablation.

**Figure 3:**
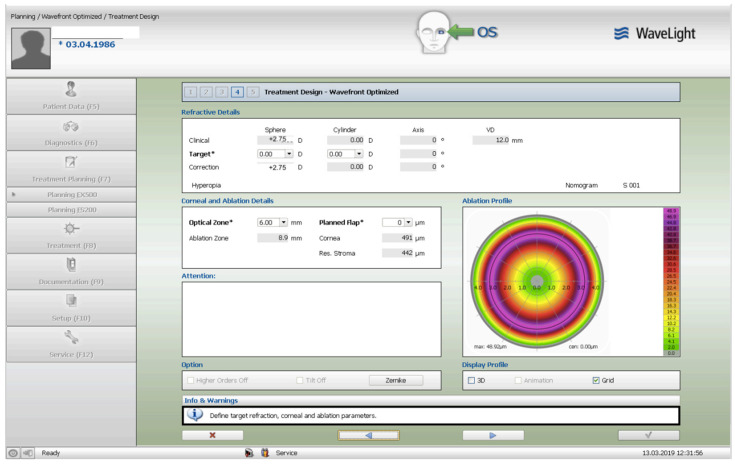
The wavefront-optimized +2.75 hyperopic treatment that was used as a second step to account for epithelial removal for a total diameter of 8.5 mm.

The treatment consisted of corneal surface excimer laser ablation aiming for normalization using a topography-guided platform (Software: Contura, Version 2.0-Alcon/Wavelight, Erlagen, Germany). A 6 mm optical zone was chosen in all cases, as seen and described in Figure 1. The epithelial removal was accounted for by adding an additional −2.75 diopter correction to the topography-guided normalizing surface ablation—seen in Figure 2. The first excimer treatment described above was followed by a second wavefront-optimized hyperopic excimer treatment of +2.75 diopters with a 6 mm optical zone, as seen in Figure 3, to account for the residual epithelial removal at an 8.5 mm diameter. The two sequential excimer ablations were followed by corneal crosslinking (CXL). This revised treatment protocol negated the use of two treatment cards—an excimer treatment necessity for all excimer lasers in the USA—one for the Contura topography-guided platform treatment. The Contura topography-guided software, version 2.0 is FDA approved only for myopic treatments in the US and used off-label in our “US Athens Protocol” described herein. The second excimer laser application was a wavefront-optimized hyperopic treatment (WFO) with the same optical zone of 6 mm. Following the two sequential ablations, we used saline-diluted riboflavin (1%) for soaking for five minutes, and then 6 mW/cm^2^ of UV light were applied for 15 min utilizing the PXL Platinum 330 device (Peschke MedTech, Germany), in continuous mode (without pulsing) and without supplemental oxygen.

All cases had the same postoperative treatment: prednisolone acetate (Pred Forte, Allergan, Irvine, CA, USA) four times a day; bromfernac ophthalmic solution (Prolensa-Bausch and Lomb, USA) for 1–2 days, depending on postoperative discomfort, up to four times a day, one drop per eye, as needed; and ofloxacin topical antibiotic (Ocuflox-Allergan, Irvine, CA, USA) four times a day until epithelialization, usually by day 5. The treatment also included Pred Forte QID for a month, followed by loteprednol ophthalmic ointment (Lotemax ointment, Bausch and Lomb, USA) used at bedtime once a day in the treated eye for an additional—the second postoperative—month.

We followed, for 36 months, for all cases with no dropouts, the refractive error, visual function, and all the other parameters, such as visual acuity, cornea clarity, keratometry, topography, pachymetry, as well as endothelial cell density. The timing of postoperative follow-ups for all cases was 1 week, 3 months, 1 year, and 3 years.

## 3. Results

**Figure 4:**
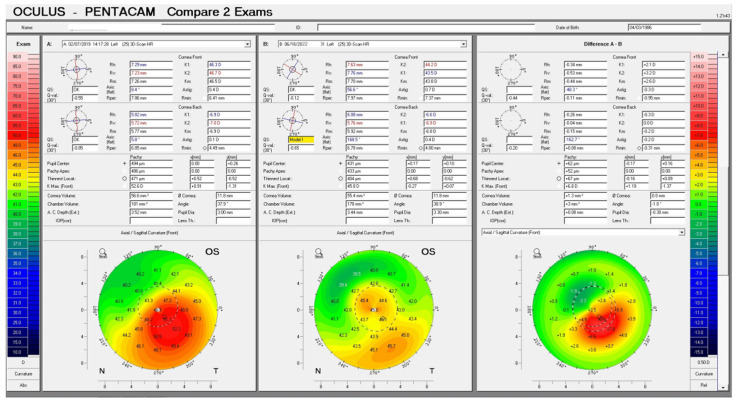
An example case of before in (**A**), after in (**B**), a difference map (**A**-**B**).

**Figure 5:**
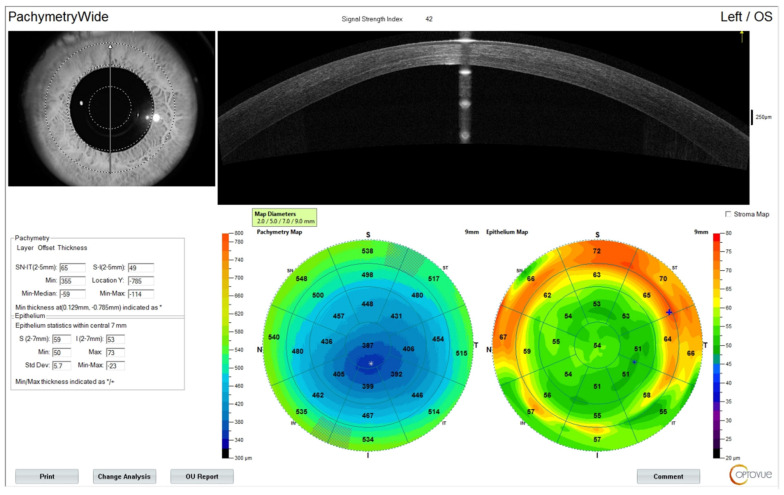
An example case imaged postoperatively after 3 months using anterior segment OCT (Avanti, Optovue, CA, USA) documenting the deep CXL line visualized in the example case at a 9 mm corneal diameter, as well as the respective epithelial remodeling—as we have reported—that may be indicative of ectasia stabilization.

The preoperative patient data and results are summarized in Table 1. Figure 1 illustrates a Contura treatment designed with zero refraction. Figure 2 illustrates that same example treatment with an additional −2.75 diopters of myopia to account for central epithelial removal as this first excimer treatment was applied on the intact epithelium. Figure 3 illustrates the wavefront-optimized +2.75 hyperopic treatment that was used as a second step to account for additional epithelial removal for a total diameter of 8.5 mm. Figure 4 depicts sequential Scheimpflug tomography assessments as an example from this case series: the preoperative axial/sagital curvature tomography map and keratometry data is shown in A, the respective postoperative results are shown in B, and the difference map, A-B (preoperative minus the postoperative), on the right. Figure 5 illustrates 3-month postoperative total corneal and epithelial thickness mapping at 9 mm diameter, as measured by anterior segment OCT (Optovue, CA, USA). The image illustrates the “deep” CXL line, as well as the epithelial remodeling that we have long reported that is indicative of the stabilization of the cornea.

The median UDVA showed marked improvement at one-year follow-up (all values in LogMAR): from 0.8 preoperative to 0.3 at 12 months, with no change at 3 years.

The median CDVA increased from 0.5 to 0.1 at 1 year. It was also stable at 3 years, at 0.1 LogMAR. The average minimal corneal thickness decreased due to the surface ablation from 466 μm to 396 μm—as recorded the first year postoperatively—and then slightly increased to 405 μm at the 3-year follow-up likely due to epithelial remodeling. The flat keratometry median value (K1) increased from 40.4 D to 42.4 D after the first postoperative year and was 41.6 D at the 3-year follow-up. The steep keratometry median value (K2) decreased from 49.5D to 44.8 D after the first postoperative year and flattened slightly more to 44.20 D at the 3-year follow-up. In accordance with these findings, the median anterior maximum keratometry (Kmax) value was 54.4 D preoperatively, flattened to 44.1 D at one year, and was 43.7 D at 3 years. The median IHD (Index of Height Decentration), as our key reported parameter of anterior corneal curvature symmetry, is tightly connected clinically, as we have reported [15], with visual performance, and the documentation of effective normalization decreased from 0.125 to 0.071 after the first postoperative year. It further changed to a median of 0.062 at the 3-year follow-up. All eyes evaluated had no further keratoconus progression at the 3-year follow-up.

There were no qualitative or quantitative endothelial cell density changes, as measured preoperatively and at 3 years postoperatively with the Konan CellCheck device (Konan, Irvane, CA, USA).

No significant complications were encountered in this group, just one case of late re-epithelization, which took 10 days but eventually progressed in a similar fashion to the cases reported herein. The absence of complications may be due to the small number of cases studied in this report.

## 4. Discussion

We have reported [12,13,14,15,22], as have many other subsequent investigators [16,17,18,19,20,21], the use of customized excimer laser ablation combined with CXL for the management of progressive keratoconus, which we have named “the Athens Protocol”.

Most subsequent modifications of this technique, globally, documented comparable outcomes, which are summarized in our recent report in the Journal of Cornea [21].

In ophthalmic practice globally, corneal crosslinking (CXL)—sometimes called by an older term, “collagen” crosslinking—emerged and has been established as a mainstream surgical intervention to stabilize progressive keratoconus and corneal ectasia [1,2,3,4,5,6,7,8,9,10,24].

Corneal stabilization has been reported to have high efficacy and safety; nevertheless, visual function, even when corneal ectasia has been stabilized, can be challenging, especially in patients intolerant to contact lens use [13].

The Athens Protocol CXL, as initially reported, comprised a surface topography-guided excimer ablation, employing—at the time—the Wavelight 200 Hz Allegretto excimer laser (Wavelight, Erlagen, Germany, later acquired by Alcon) and was described initially as “partial” PRK (photorefractive keratectomy), where “partial” refers to the manifest subjective refraction of each case. In essence, only 1 to 2 diopters of spherocylindrical treatment was included, which served more as a phototherapeutic keratectomy (PTK), customized in diameter, transition zone, and refractive error—if any was added—to regularize the ectasia-induced irregular corneal power refractive changes that were associated with the respective loss of functional visual acuity and visual function.

We have previously reported the comparison with alternative reported clinical protocols [22].

This approach carried an obvious disadvantage when applied after the CXL procedure at a later time—besides the stromal thinning noted above—the actual removal of cross-linked stroma. In CXL for ectasia, the anterior stroma appears to become biomechanically “stronger”, and this biomechanical effect diminishes in the inner deeper stromal layers [1,2,3,4].

The initial outcomes reported were compelling: keratoplasty was essentially not anymore indicated as robust visual rehabilitation was achieved, in some cases even without refractive correction [12,13,14,15].

In essence, the same-day combined CXL–therapeutic PRK technique, termed “the Athens Protocol”, targeted the structural stabilization of corneal ectasia while augmenting in multiplicity the refractive normalization and function by significantly optimizing the central 5 mm anterior corneal curvature. This approach was unable—since the ablation was mainly based on surface reflection topography data—to address posterior corneal irregularities, mainly coma.

The common primary objective among all clinicians attempting this combined customized surface ablation with CXL was also to minimize excimer stromal tissue ablation-by customizing the therapeutic surface ablation to mainly address irregular astigmatism and central refractive coma.

All reports support that these corneal changes have been pivotal in the improvement of uncorrected and corrected distance visual acuity (UDVA and CDVA, respectively), as well as the reduction of keratometry asymmetry indices [12,13,14,15,16,17,18,19,20,21,22,23,25,26,27,28,29,30,31,32].

## 5. Athens Protocol CXL Technique Principles and Potential Advantages

As introduced, a limit of stromal tissue removal using excimer laser ablation from the thinnest corneal area was set at 50 μm, as measured initially by scanning-slit tomography and later by Scheimpflug and/or OCT tomography, a limit that has been adapted by most subsequent technique modifications [12].

Customized corneal surface ablation was topography-guided by most investigators, while wavefront-guided by a few others [12,13,14,15,16,17,18,19,20,21,22,23,25,26,27,28,29,30,31,32].

Topography-guided customization of the excimer ablation is driven by topography captured data, and, even when set to correct no refractive error, it provides an ablation algorithm attempting to “flatten” the corneal areas most steepened by ectasia and “steepen” the adjacent areas—usually the superior most flattened corneal area within the central 5 to 6.5 mm of the cornea. This concept is best illustrated in Figure 1.

Such “therapeutic” excimer surface ablations combined in the same session with CXL mostly employ higher fluence UV irradiance for a shorter time exposure, commonly termed “accelerated” CXL, for which the total energy delivered is usually 5.5 to 7 Joules. It is therefore invariably “epithelium-off”, as even Bowman’s membrane has been ablated by the surface ablation prior to the riboflavin solution soaking and the UV light exposure. A minimum stromal thickness of 400 μm is pursued, although we have reported in advanced cases, effective treatment up to a 380 m minimum total corneal thickness, measured prior to epithelial removal [12,13,14,15,16,17,18,19,20,21,22,23,24,25,26,27,28,29,30,31,32].

Apart from the obvious advantages of the combined CXL + PRK of the Athens Protocol technique and its modifications having the efficiency of completing the operative procedure in one day with a single postoperative period, other basic reported benefits include the following:

Higher efficacy of central cone flattening combined with respective superior cornea steepening, surpassing the overall central corneal curvature normalization as would be predicted by sequential CXL and PRK, achieving sustained stability even in over 10 years follow-up [13].
A uniform, “deeper” CXL effect extending both deeper, to 60–80% of the residual stromal thickness, and wider, up to a 9 mm corneal diameter, as evident from slit lamp biomicroscopic analysis as well as the anterior segment OCT “CXL demarcation line”.
Less postoperative scarring associated with combined vs. sequential protocol cases—we found this to be significantly reduced by maximizing the transition zone of the topography-guided ablation [22].

Excimer ablation of a thinned cornea undergoing ectasia remains a serious concern because of the potential risk of biomechanically weakening the residual stream bed, which thereby increases the potential risk for further ectasia. Yet, the long-term combined procedure results fortunately do not identify such concerns as corneal curvatures remain flattened and normalized (reductions ranging from 8 to 20 diopters), suggesting a synergy between “cone-flattening” and corneal curvature normalization, presumably consequent to the “deeper” and “broader” CXL effect. A minimum stromal thickness of 350 μm was suggested as the lower limit for performing this approach [12], necessitating respective adjustment of the “partial”—in regard to the refractive error targeted—corneal surface ablation, which is not to exceed this planned lower limit.

Several long-term reports review the technique outcomes and further elaborate on the key technique steps [11,15,16,22,23,24,25,26,28,29,30,31,32].

In summary, the preponderance of published evidence supports combined CXL “plus” customized minimal tissue ablation PRK to be a safe and effective strategy, long term, for anatomical and visual management of keratoconus and post-LASIK ectasia.

Although almost half of treated eyes remain variably ametropic—as the ablation limitations described above cannot address the full refractive error—most cases have increased the ability to employ visual rehabilitation with spectacles and/or contact lenses, or even subsequent phakic IOL implantation [29]. Additionally, improved soft contact lens comfort tolerance and even, in some cases, rigid gas-permeable contact lens comfort tolerance following CXL have been reported as CDVA demonstrates visual improvement.

Complications associated with the combined CXL + PRK technique and the management thereof have been reported [30]:

Delayed corneal re-epithelization beyond that anticipated for a PRK procedure. To promote more efficient re-epithelialization, the reduction of topical corticosteroids and the addition of autologous platelet-rich plasma [20] are beneficial.
Cornea stromal haze over the ablation areas can develop as late as a year post-procedure in pediatric patients, possibly in response to intense natural UV light exposure.
Potential deep stromal scarring associated with CXL, as evident from slit lamp biomicroscopic analysis and anterior segment OCT, is not specific to Athens Protocol procedures and, rather, should be carefully observed and managed during the first 2 months following the procedure.
A potential progressive additional flattening effect developing years after treatment has been reported in <2% of cases. This may result in significant hyperopic shift requiring revision of optical visual correction and/or additional refractive surgical intervention.

Residual stromal pachymetry is a principle of precaution and concern:

The greatest limitation and safety consideration is the limit of PRK tissue removal in an already thinned cornea, which, as mentioned above, we desire to be over 400 μm (total corneal thickness at the thinnest point) prior to the partial thickness surface ablation, a concern which also merits an informed consent discussion of the risks, benefits, and alternatives. Based on this concern, we sometimes limit the surface ablation to solely epithelial removal using the excimer laser (50 μm depth, 7 mm diameter), where in areas of epithelial thinning to <50 μm, there is also some Bowman’s layer and underlying stroma selective ablation that can enhance the effect of CXL. For example, if at the “peak” of the cone the epithelium has remodeled to 30 μm, a 50 μm PTK will remove the 30 μm of epithelium and the respective 10 μm of the Bowman’s layer—although, in these advanced cases, it may be significantly thinner and the residual ablation thickness, to 50 μm, includes the corresponding anterior stroma. As noted above, a minimum residual stromal thickness of 350 μm is targeted following the partial thickness surface ablation, and, in advanced cases, up to 330 μm is targeted [14] as CXL in thinner stromal situations may not stabilize enough corneal stromal “volume” and, as a second concern, the UV penetrance during the procedure may become cytotoxic to the corneal endothelium.
Another potential CXL modification is the customized application of the UV irradiation at variable fluence and variable pattern profiles, thereby utilizing CXL as an enhanced flattening tool when compared to standard CXL with uniform UV light application [23].
Continued eye-rubbing appears to be a pivotal factor in the mechanism of ectasia development: it is now likely that eye rubbing, even during sleep, is one of the pivotal activities that contribute to the development and progression of keratoconus and cornea ectasia. Thus, proper education and continued reinforcement of eye-rubbing avoidance can be highly beneficial in ectasia stabilization and long-term prognosis [22,24,32].
The most studied and utilized platform for topography-guided corneal reshaping addresses the anterior corneal curvature. Thus, visual rehabilitation is strongly influenced by the actual cone location regarding the cornea center. Severely oblique cones will significantly normalize with this technique regarding anterior cornea curvature but will still retain irregular posterior curvature that can functionally limit vision. Wavefront-guided or ray-tracing customization of the therapeutic surface ablation used in the Athens Protocol CXL and modified techniques have been reported to address this point. The potential advantages noted are the measurement and potential normalization of the total corneal high-order aberrations, with less tissue removal planned over the thinnest cone area as raytracing appears to address a typical inferior corneal ectasia because of the corneal tilt about the total eye refractive system, additionally addressing the refractive role of both the anterior and posterior corneal curvature [22].

As cornea cross-linking has changed the management paradigm for keratoconus, revisiting the keratoconus diagnostic criteria has become increasingly crucial.

Utilizing modern corneal diagnostics such as Scheimpflug corneal tomography and anterior segment OCT corneal tomography and epithelial mapping, as well as high frequency ultrasound corneal epithelial mapping, early signs of ectasia become detectable, even when visual function, slit lamp biomicroscopic analysis, and traditional corneal topography appear normal [22]. Cornea epithelial remodeling may be able to mask traditional topographic imaging signs in early corneal ectasia, as well as explain corneal topographic steepening that is not associated with ectasia progression but rather local epithelial thickening of the cone apex.

Recent reports support the consideration of keratoconus screening in first- and second-degree family members as becoming crucial in early diagnosis. As prevention is—in so many medical contexts—the best medicine, early detection of keratoconus and other ectasias affords the best opportunity for arresting progression and optimizing vision in the earliest stages of these important corneal conditions [22].

We introduce herein this novel technique utilizing the approved and available parameters for a specific excimer laser within the United States. As noted above, it is an off-label application for the US, enabling a similar treatment protocol to the Athens Protocol. No postoperative problems were encountered within this small group of cases.

These data essentially “mirror” the European experience with the Athens Protocol, as well as data published with similar and modified techniques globally [11,12,13,14,15,16,17,18,19,20,21], offering clinical reference for fellow surgeons in the United States, given that the appropriate informed consent is provided to each patient, as this application is “off-label”.

Although several clinicians still opt to perform cross-linking first, to be followed by topography-guided surface ablation as a therapeutic normalization process later postoperatively—despite the fact that we have established in the literature the advantage that both being used may offer a synergistic effect that is multiple times more effective for cornea normalization than if you use the two techniques at separate times—the potential disadvantage of performing surface ablation after the initial CXL procedure is the removal of some of the most “cross-linked” anterior stromal tissue by the initial CXL procedure [12,13].

Despite the similar clinical data, one of the limitations of the US-allowed parameters is the lower limit of a 6 mm optical ablation zone, which cannot be decreased to 5 mm—the minimum available with this technology outside the US—thus posing a limitation in the spectrum of some advanced ectasia cases, which due to significant thinning prohibit the application of this type of treatment.

Theoretically, when corneal curvature normalization is the priority, and not necessarily reducing the amount of myopia in these corneas with advanced ectasia, utilizing the hyperopic WFO treatment aimed at epithelial removal only could bypass this tissue limitation at the cost of inducing a more myopic refractive error. For example, if a 400-micron cornea is encountered as the minimum cornea thickness and the Athens Protocol CXL treatment has decided—or is speculated to be applied—then the actual topography-guided treatment can be performed on the intact epithelium without adding the −2.75 D factor for epithelial removal as described above. Then, the hyperopic WFO treatment for epithelial removal can follow. As a result, the cornea will be normalized, but the actual end-refractive error will have a myopic shift.

In essence, the postoperative refraction will be expected to become more myopic than preoperatively, nevertheless with parallel expected improvement in the CDVA that may be corrected with spectacles, contact lenses, and even phakic IOL later.

Further studies of this concept may further validate these initial clinical data, and the approval of customized treatments that may address high-order aberrations not limited to the cornea may enhance the potential options for surface excimer treatment.

## Figures and Tables

**Table 1:** Patient Demographic and Preoperative Refraction and Keratometry Data.

Sex: 12 Male, 5 Female. Median Values: Age	30 Years (Range: 21–49)	
Spherical Equivalent	Median: −5 D (range: −1.25 to −9.75)	
Astigmatism	Median: −2.75 D (range: −1.25 to −9.75)	
Keratometry Steep	Range: 41 to 56.5 D	Median: 49.5 D
Keratometry Flat	Range 39.9 to 45.5 D	Median: 40.4 D

D = diopters.

## Data Availability

Data are contained within the article. No other Data are unavailable due to privacy restrictions.
